# IL-10 polymorphisms *+434T/C*, *+504G/T*, *and -2849C/T* may predispose to tubulointersititial nephritis and uveitis in pediatric population

**DOI:** 10.1371/journal.pone.0211915

**Published:** 2019-02-19

**Authors:** Sari Rytkönen, Jarmo Ritari, Juha Peräsaari, Ville Saarela, Matti Nuutinen, Timo Jahnukainen

**Affiliations:** 1 PEDEGO Research Unit and Medical Research Center (MRC), University of Oulu and Oulu University Hospital, Oulu, Finland; 2 Clinical Laboratory, Finnish Red Cross Blood Service, Helsinki, Finland; 3 Ville Saarela, Department of Ophthalmology, Oulu University Hospital, Finland; 4 Timo Jahnukainen, Department of Pediatric Nephrology and Transplantation, New Children’s Hospital, University of Helsinki and Helsinki University Hospital, Helsinki, Finland; Tor Vergata University, ITALY

## Abstract

**Background:**

Tubulointerstitial nephritis (TIN) and uveitis syndrome (TINU) are likely to be autoimmune diseases. Based on previous studies, adults with isolated idiopathic uveitis have polymorphisms in interleukin 10 (IL-10) and tumor necrosis factor α (TNF-α) genes. We aimed to evaluate the presence of IL-10 and TNF-α polymorphisms in a nationwide cohort of pediatric TIN/TINU patients.

**Methods:**

Single nucleotide polymorphisms in IL-10 (*+434T/C*, *+504G/T*, *-1082G/A*, *-2849C/T*) and in TNFα (*-308G/A*, *-238G/A*, *-857C/T*) genes were genotyped in 30 well-defined pediatric patients with idiopathic TIN/TINU syndrome. Control group frequencies for these SNPs were obtained from 393 independent Finnish subjects.

**Results:**

The homozygous minor allele in *IL-10 +434T* (rs2222202) and IL-10*+504G* (rs3024490) was found in all patients with TIN or TINU syndrome while the frequency of these minor alleles in the control population was 44% and 23%, respectively (*p* <0.001). In IL-10 SNP -2849 (rs6703630) a significant difference was found with genotype TT in all patients (*p* = 0.004) and in subgroups with TINU syndrome (*p* = 0.017) and TINU syndrome with chronic uveitis (*p* = 0.01) compared to reference population. There were no statistical differences in any of the studied TNF-α genotypes between TIN/TINU patients and control population.

**Conclusions:**

A significant difference in the frequency of *IL-10+434T* and +*504G* alleles was found between TIN/TINU patients and control population. Genotype -2849TT was more frequently present in patients with TINU syndrome than in the reference subjects. Genetic variation in the inflammatory mediators may predispose to autoimmune nephritis and uveitis.

## Introduction

Tubulointerstitial nephritis (TIN) is a relatively rare but significant cause of acute renal insufficiency (AKI) among children and adults [1]. It is an inflammatory disease, possibly of autoimmune origin [2–5], primarily affecting the renal interstitium and tubular wall without significant glomerular or vascular involvement [6,7]. It can sometimes be accompanied by uveal inflammation (TINU syndrome) which is typically anterior and bilateral [8, 9].

TIN may be triggered by several causes including infections and medications, or etiology can be idiopathic [10]. TIN and uveitis separately can also be associated with systemic immunologic conditions such as sarcoidosis, systemic lupus erythematosus (SLE) or inflammatory bowel disease (IBD) [11–13]. Previous studies have shown evidence of polymorphisms in interleukin 10 (IL-10) and tumor necrosis factor α (TNF-α) coding genes in patients with non-infectious uveitis (NIU) [14, 15]. There are also recent data showing that polymorphisms in these two inflammatory regulators are enriched in patients with inflammatory bowel disease and children with wheezing [16–19]. In this study, the aim was to investigate the frequency of IL-10 and TNF-α single nucleotide polymorphisms (SNPs) in a national cohort of well-defined children and adolescents with TIN/ TINU syndrome compared to Finnish reference population.

## Materials and methods

The study was approved by the Ethics Committee of Helsinki University Hospital and followed the tenets of the Declaration of Helsinki. Written informed consent was obtained from all patients and/or parents before the study commenced.

This study was part of our previous nationwide study evaluating HLA associations in TIN/TINU patients [9, 20] and the patient demographics has been reported before. Briefly, the inclusion criteria were biopsy-proven TIN in pediatric patients under 16 years of age. The study cohort was collected from all five university hospitals in Finland between 2008 and 2011. Meticulous work-up was done to exclude possible underlying conditions, such as drug-induced TIN, respiratory infection, sarcoidosis, connective tissue disorder and lymphoma. Uveitis was classified according to standardization of uveitis nomenclature (SUN) criteria. All patients were followed up by a pediatric nephrologist and ophthalmologist for at least one year after the diagnosis of TIN [9, 20, 21].

A total of 30 patients were enrolled (17 boys, 13 girls). Nineteen patients (63%) had uveitis (TINU syndrome) and 15 (50%) of them had chronic uveitis. The median age at the time of diagnosis was 12.5 (9.4–14.7) years. Demographic data is presented in Table 1.

**Table 1:** Patient demographics and key laboratory findings at the time of the diagnostic biopsy. Data is presented for all patients and for males and females separately.

	All patients	Females	Males	*p*-value*
Patients, n (%)	30 (100)	13 (43)	17 (57)	
Age, years	12.5 (9.4–14.7)	12.3 (11.4–14.8)	11.9 (9.1–15.4)	0.834
TIN, n (%)	11 (37)	4 (31)	7 (41)	0.564
TINU, n (%)	19 (63)	9 (69)	10 (59)	0.564
Chronic TINU, n (%)	15 (50)	6 (46)	9 (53)	0.717
CRP, mg/L	26 (4–53)	36^a^ (4–54)	17 ^a^(4–64)	0.439
ESR, mm/h	92 (38–108)	92^b^ (54–109)	73^b^ (23–98)	0.204
P-Crea, μmol/L	189 (136–358)	209 (151–400)	167 (94–209)	0.676
GFR, ml/min/1.73m^2^	44 (23–57)	39 (17–47)	53 (35–76)	0.451

TIN, Tubulointerstitial nephritis; TINU, tubulointerstitial nephritis with uveitis syndrome; CRP, C-reactive protein; P-Crea, plasma creatinine concentration; ESR, erythrocyte sedimentation rate; GFR, glomerular filtration rate. Values in the table are presented as median with the interquartile range in parenthesis.

* *P*-value calculated for difference between females and males, Mann-Whitney U -test.

^a^ CRP values available, females n = 7, males n = 11

^b^ ESR values available, females n = 10, males n = 11

The targeted single nucleotide polymorphisms from our TIN patient cohort were IL-10 *+434T/C*, *+504G/T*, *-1082G/A*, *-2849C/T* and TNF-α *-308G/A*, *-238G/A*, *-857C/T*. The primers used in polymerase chain reaction (PCR) to amplify DNA fragments containing our SNPs of interest are presented in Table 2. Genomic DNA samples were sequenced in Oulu University DNA sequencing core facility laboratory with the following method: The PCR amplifications were carried out in a total volume of 11μL, which contained 20 ng genomic DNA, 25 mM MgCl2, 2 mM dNTP mix, 5 μM of each primer, 0.4 unit Maxima Hot Start Taq DNA polymerase and 1 x Hot Start PCR Buffer (Fermentas). PCR products were screened using QIA excel Advanced System (QIAGEN) to confirm the successful amplification. Agencourt AMPure protocol (Beckman Coulter) was used to purify PCR products for sequencing. Finally, the samples were sequenced using ABI3500xL Genetic Analyzer and BigDye Terminator vs.1.1 reagents (Life Technologies). Chromatograms were analyzed by using the Chromas Lite 2.1 program.

**Table 2:** Primers used in polymerase chain reaction to amplify DNA fragments.

SNP	Primers	
IL-10		
-2849 (rs6703630)	reverse	5´-GGCCCGGATCTGACTTCTTT-3´
-1082 (rs1800896)	reverse	5´-TAAACTTTAGACTCCAGCCACAG-3´
+434 (rs2222202)	forward	5´-ATCCCCAACACCTATTCCCC-3´
+504 (rs3024490)	forward	5´-AAATGCGTTCTCTCTCGTGC-3´
TNFα		
-308 (rs1800629)	reverse	5´-CCCAACTTTCCAAATCCCCG-3´
-238 (rs361525)	reverse	5´-TACCGCTTCCTCCAGATGA-3´
-857 (rs1799724)	reverse	5´-GGGGCCCTGAGAAGTGAG-3´

IL-10, Interleukin10; TNFα, Tumor necrosis factor α

The frequency of these SNPs was analyzed in the whole study population and in the subgroups of patients with TIN, TINU and TINU with chronic uveitis. Control group (*n* = 686) frequencies for the IL-10 and TNF-α SNP were obtained from both Illumina Immunochip [22] analysis of 587 Finnish siblings and from the 1000 Genomes Project Finnish population subset (*n* = 99) [23]. The Immunochip hybridization and genotype calling were performed at the Institute for Molecular Medicine Finland (Helsinki, Finland) according to manufacturer’s instructions. Since the TNF-α SNP -857 / rs1799724 and IL-10 SNP -2849 / rs6703630 were not included in the Immunochip array, their frequencies were imputed using Impute2 v3.2.3 software [24] with default settings and 1000GP_Phase3 as the reference data set. The imputed sequence intervals in GRCh37 Human genome build were 206000000–208000000 for chromosome 1 (rs6703630) and 30500000–32600000 for chromosome 6 (rs1799724).

## Statistical analysis

Statistical analyses were carried out with R v3.2.2 (R Core Team 2015). Fisher’s exact test was performed for significance of association for SNP frequencies between patients and controls. The reference SNP frequencies used in Fisher’s test were weighted by the respective sample sizes from the Immunochip (n = 587) and 1000 Genomes (n = 99) data sets. Since the Immunochip data consisted of sibling pairs, the sample size number used in Fisher’s test was halved to take into account the fact that relatedness reduces the effective sample size. Logistic regression was performed for SNP genotype association using the Immunochip sibling cohort as a reference. Finally, all raw p-values were adjusted for multiple testing by the Bonferroni method.

## Results

The homozygous minor allele in *IL-10 +434T* (rs2222202) and IL-10*+504G* (rs3024490) was found in all patients with TIN/TINU syndrome (Table 3). The frequency of these minor alleles in the control population was 44% and 23%, respectively (*p* <0.001). The presence of minor allele in IL-10 *-2849T* did not differ significantly between the cases and the controls (*p* = 0.33) (Table 3). However, the homozygous *-2849TT* genotype was found more frequently (p = 0.004) in the patients (17%) than in the controls (0.01%) (Table 4). There were no statistical differences in any of the studied TNF-α alleles between TIN/TINU patients and control population (Table 3, S1 Table).

**Table 3:** Distribution of alleles in single nucleotide polymorphisms between study population and control subjects. The genotypes frequencies are presented in TIN/U patients.

SNP locus	Allele	Patients % 2n = 60	Controls %		*p* value	Genotype %	
			2n = 785*				
			IC 1000Genomes				
IL-10–2849	C	32	83	83	0.33	CC	53
(rs6703630)	T	68	17	17		CT	30
						TT	17
IL-10–1082 (rs1800896)	C	48	46	40	1.00	TT	30
	T	52	54	60		CC	27
						TC	43
IL-10 +434 (rs2222202)	T	100	46	40	<0.001	TT	100
	G	0	54	60		TG	0
						GG	0
IL-10 +504 (rs3024490)	G	100	78	76	<0.001	GG	100
	A	0	22	24		AG	0
						AA	0
TNFα -308	G	87	89	87	1.00	GG	86
(rs1800629)	A	13	11	13		AGG	14
						AA/AG	0/0
TNFα -238	G	100	98	96	1.00	GG	100
(rs361525)	A	0	2	4		AG	0
						AA	0
TNFα -857	C	87	92	93	1.00	CC	87
(rs1799724)	T	13	8	7		TC	13
						TT	0

SNP, Single nucleotide polymorphism; Il-10, Interleukin 10; TNFα, Tumor necrosis factor α. p-values are adjusted for multiple testing by the Bonferroni- method.

* Illumina Immunochip analysis of 587 Finnish siblings (IC) and 1000 Genomes project, Finnish population subset n = 99

**Table 4:** Significant differences in IL-10 minor alleles and genotypes between study cohort and control population. Data is presented for all patients and depending on the presence and chronicity of uveitis.

SNP locus	Minor allele	P value	P value	P value	P value
		All pts, 2*n* = 60	TIN, 2n = 22	TINU, 2n = 38	Chronic uveitis, 2n = 30
IL-10–2849 (rs6703630)	T	0.33	1.00	1.00	1.00
OR^†^		2.3	2.68	2.14	2.36
(95% CI)		(1.2–4.2)	(0.89–7.40)	(0.96–4.47)	(0.92–5.62)
IL-10 +434 (rs2222202)	T	<0.001	<0.001	<0.001	<0.001
IL-10 +504 (rs3024490)	A	<0.001	0.50	0.004	0.07
	Genotype	P value	P value	P value	P value
		All pts, n = 30	TIN, n = 11	TINU, n = 19	Chronic uveitis, n = 15
IL-10–2849 (rs6703630)	CT*	1.00	1.00	1.00	1.00
	TT^#^	0.004	0.275	0.017	0.01

OR, Odds ratio; SNP, Single nucleotide polymorphism; Pts, Patients; TIN, Tubulointerstitial nephritis; TINU, Tubulointerstitial nephritis with uveitis syndrome; IL-10, Interleukin 10; CI, Confidence interval. Control group for allele frequency 2n = 785. Control group for genotype frequency consist only of Illumina Immunochip population, n = 257; Genotype frequency analysis was done with logistic regression; p-values are adjusted for multiple testing by the Bonferroni method.

*heterozygous patients n = 9/30% vs. controls 21%

^#^homozygous patients n = 5/17% vs. controls 0.01%

†Odds ratio for minor allele frequency was calculated when applicable. No OR for SNPs IL-10 +434 and IL-10 +504 were calculated because the other allele frequency was zero.

In the subgroup analysis, a significant increase in the allele frequency of IL-10*+434T* (rs2222202) was found in patients with isolated nephritis (*p* <0.001), TINU syndrome (*p* <0.001) and TINU with chronic uveitis (*p* <0.001) when compared to the reference population (Table 4). IL-10*+504G* (rs3024490) minor allele was present more often in TINU patients (*p* = 0.004) but not in subgroups with isolated TIN (p = 0.5) or TINU syndrome with chronic uveitis (p = 0.07) (Table 4).

In SNP -2849 (rs6703630) a significant difference in genotype frequency TT was found in patients with TINU syndrome (*p* = 0.017) and TINU syndrome with chronic uveitis (*p* = 0.01) when compared to the reference population (Table 4).

## Discussion

In the present study, we aimed to investigate the frequency of IL-10 and TNF-α polymorphisms in a cohort of Finnish pediatric patients with idiopathic TIN/TINU syndrome. All patients with either isolated nephritis or TINU syndrome were homozygous carriers of the IL-10 *+434T* and *+504G* minor alleles, which suggests that these SNPs may predispose to TIN and/or TINU. In addition to IL-10 *+434T* and *+504G* minor alleles, the patients with uveitis and chronic uveitis had significantly more frequently IL-10 *-2849TT* genotype than Finnish control population. Despite the high occurrence of uveitis in the present study population, none of the previously reported TNF-α SNPs *-308G/A*, *-238G/A*, *-857C/T* were found in this cohort. Our results suggest that IL-10 polymorphisms may have a role in susceptibility to TIN/TINU while genetic variation in TNF-α gene may be connected to isolated uveitis.

It is obvious that tendency for uveitis with or without tubulointerstitial nephritis is dependent on genetic factors. We, among others, have previously shown an association between HLA haplotypes and uveitis and/or TIN [2, 3, 25, 26]. Based on our present findings, variability in the IL-10 gene may predispose to TIN/TINU in pediatric population. IL-10 production is stimulated by various exogenous and endogenous factors, but it has also been shown to be under genetic control with association to different SNPs in several independent studies [27–29]. Different allele and genotype variations alter cytokine profiles influencing the development and severity of various diseases, as documented in SLE, tuberculosis and several other infectious diseases (IL-10 overexpression) [30–36], and rheumatoid arthritis and asthma (IL-10 downregulation) [19, 37–39]. Polymorphisms in inflammatory response regulating genes have been reported among patients with uveitis [40–42]. Atan et al. have previously shown a significant association between IL-10–*2849*, *+434*, *+504* minor allele frequencies and non-infectious uveitis (NIU) in a cohort of 192 patients [14]. The SNPs *+434* (rs2222202) and *+504* (rs3024490) located in introns are in regulatory regions, and may influence the expression of mRNA and/or protein. The same point is related to SNP*-2849* (rs6703630) located in the 5'UTR region. According to the Genotype-Tissue Expression (GTEx) database (https://gtexportal.org), IL-10 mRNA expression is increased significantly in whole blood samples from human subjects with minor allele SNPs *+434* (rs2222202), *+504* (rs3024490), and *-2849* (rs6703630). This data indicates, that these homozygous minor alleles have impact on gene regulation separately, however, their cooperative action and influence on IL-10 level remain unclear.

In the present study, previously reported, association between TNF-α alleles *-308AA* and *-238AA*, IL-10 SNP *-1082AA* and TIN/TINU could not be found [14, 15]. All patients, including those with chronic uveitis, had *-238GG* and *-308GG* genotype, which suggests that in TIN patients the studied genetic variation in TNF-α does not have any influence on uveitis risk. The genetic background in TIN/TINU patients is probably different from patients with isolated uveitis. This is also supported by our previous finding in this same study population showing that none of the patients had the HLA-DRB1*15 genotype, which has been suggested to be associated with increased risk for uveitis [43, 44] and is relatively common in the Finnish population (15%) [3]. It is also likely, that the association between HLA genotype as well as genetic variation in cytokine genes and uveitis appear to depend on study population and ethnicity [45]. In previous studies, the patient cohorts have been rather heterogeneous consisting of patients with varying causes of uveitis, such as sarcoidosis, Bechet’s disease, sympathetic ophthalmia, intermediated uveitis, white dots with or without inflammation, and the age range of the patients was wide. [14, 15]. In the present study, all patients had biopsy proven nephritis and systemic diseases and infectious agents were excluded. In addition, the patients were prospectively followed-up by pediatrician and ophthalmologist at least 12 months from initial diagnosis, which is likely to improve the reliability of our findings. It is also of note that in the present study, all patients had TIN and approximately half of them had chronic uveitis. The presence of nephritis has not been reported in majority of the previous studies.

The major caveat of the study is the relatively small study population. TIN/TINU is a relatively rare entity. We have collected patients from all university hospitals in Finland and this is one of the largest pediatric data that has been published. In addition, all patients were evaluated carefully and followed up prospectively using the same protocol. Another weakness is that we did not measure serum levels of IL-10 and TNF-α, and therefore based on our data, we are not able to draw any conclusions about the clinical relevance of the identified SNPs in this study population.

In conclusion, IL-10 gene polymorphisms +434T/C, +504G/T, and genotype -2849TT were found in a majority of the TIN/TINU patients while the frequency of none of the previously reported TNFα SNPs differed from control population. The clinical importance of this finding remains to be studied.

## Supporting information

S1 Table Clinical information and TNFa -308, TNFa -238, TNFa -857, IL10–2849, IL10–1082, IL10 +434, IL10 +504 genotype of each patient. (XLSX)

## References

[1] Greising J, Trachtman H, Gauthier B, Valderrama E. Acute interstitial nephritis in adolescents and young adults. Child Nephrol Urol 1990;10: 189–195. 2088589

[2] Levinson RD, Park MS, Rikkers SM, et al Strong associations between specific HLA-DQ and HLA-DR alleles and the tubulointerstitial nephritis and uveitis syndrome. Invest Ophthalmol Vis Sci. 2003;44: 653–657. 12556395 10.1167/iovs.02-0376

[3] Peräsaari J, Saarela V, Nikkilä J, et al HLA associations with tubulointerstitial nephritis with or without uveitis in Finnish pediatric population: a nation-wide study. Tissue Antigens. 2013;81: 435–441. 10.1111/tan.12116 23594347

[4] Reddy AK, Hwang YS, Mandelcorn ED, Davis JL. HLA-DR, DQ class II DNA typing in pediatric panuveitis and tubulointerstitial nephritis and uveitis. Am J Ophthalmol. 2014;157: 678-686.e671-672. 10.1016/j.ajo.2013.12.006 24321473

[5] Rytkönen SH, Kulmala P, Autio-Harmainen H, et al FOXP3+ T cells are present in kidney biopsy samples in children with tubulointerstitial nephritis and uveitis syndrome. Pediatr Nephrol. 2018;33: 287–293. 10.1007/s00467-017-3796-z 28894974

[6] Ellis D, Fried WA, Yunis EJ, Blau EB. Acute interstitial nephritis in children: a report of 13 cases and review of the literature. Pediatrics. 1981;67: 862–870. 7015263

[7] Takemura T, Okada M, Hino S, et al Course and outcome of tubulointerstitial nephritis and uveitis syndrome. Am J Kidney Dis. 1999;34: 1016–1021. 10.1016/S0272-6386(99)70006-5 10585310

[8] Mandeville JT, Levinson RD, Holland GN. The tubulointerstitial nephritis and uveitis syndrome. Surv Ophthalmol. 2001;46: 195–208. 11738428 10.1016/s0039-6257(01)00261-2

[9] Saarela V, Nuutinen M, Ala-Houhala M, Arikoski P, Rönnholm K, Jahnukainen T. Tubulointerstitial nephritis and uveitis syndrome in children: a prospective multicenter study. Ophthalmology. 2013; 120: 1476–1481. 10.1016/j.ophtha.2012.12.039 23511116

[10] Praga M & González E. Acute interstitial nephritis. Kidney Int. 2010;77: 956–961. 10.1038/ki.2010.89 20336051

[11] Coutant R, Leroy B, Niaudet P, et al A Renal granulomatous sarcoidosis in childhood: a report of 11 cases and a review of the literature. Eur J Pediatr 1999;158: 154–159. 10048615 10.1007/s004310051038

[12] Oikonomou K, Kapsoritakis A, Eleftheriadis T, Stefanidis I, Potamianos S. Renal manifestations and complications of inflammatory bowel disease. Inflamm Bowel Dis. 2011;17: 1034–1045. 10.1002/ibd.21468 20842645

[13] Pagni F, Galimberti S, Galbiati E, et al Tubulointerstitial lesions in lupus nephritis: International multicentre study in a large cohort of patients with repeat biopsy. Nephrology (Carlton). 2016;21: 35–45. 10.1111/nep.12555 26132414

[14] Atan D, Fraser-Bell S, Plskova J, et al Cytokine polymorphism in noninfectious uveitis. Invest Ophthalmol Vis Sci. 2010;51: 4133–4142. 10.1167/iovs.09-4583 20335604

[15] Atan D, Heissigerova J, Kuffová L, et al Tumor necrosis factor polymorphisms associated with tumor necrosis factor production influence the risk of idiopathic intermediate uveitis. Mol Vis. 2013;19: 184–195. 23378732 PMC3559088

[16] Amre DK, Mack DR, Morgan K, et al Interleukin 10 (IL-10) gene variants and susceptibility for paediatric onset Crohn's disease. Aliment Pharmacol Ther. 2009;29: 1025–1031. 10.1111/j.1365-2036.2009.03953.x 19210299

[17] Bilolikar H, Nam AR, Rosenthal M, Davies JC, Henderson DC, Balfour-Lynn IM. Tumour necrosis factor gene polymorphisms and childhood wheezing. Eur Respir J. 2005;26: 637–646. 10.1183/09031936.05.00071104 16204594

[18] Ferguson LR, Huebner C, Petermann I, et al Single nucleotide polymorphism in the tumor necrosis factor-alpha gene affects inflammatory bowel diseases risk. World J Gastroenterol. 2008;14: 4652–4661. 10.3748/wjg.14.4652 18698679 PMC2738789

[19] Korppi M, Nuolivirta K, Lauhkonen E, et al P IL-10 gene polymorphism is associated with preschool atopy and early-life recurrent wheezing after bronchiolitis in infancy. Pediatr Pulmonol. 2017;52: 14–20. 10.1002/ppul.23489 27228545

[20] Jahnukainen T, Ala-Houhala M, Karikoski R, et al Clinical outcome and occurrence of uveitis in children with idiopathic tubulointerstitial nephritis. Pediatr Nephrol. 2011;26: 291–299. 10.1007/s00467-010-1698-4 21120539

[21] Jahnukainen T, Saarela V, Arikoski P, et al Prednisone in the treatment of tubulointerstitial nephritis in children. Pediatr Nephrol. 2013;28: 1253–1260. 10.1007/s00467-013-2476-x 23605375

[22] Cortes A & Brown MA. Promise and pitfalls of the Immunochip. Arthritis Res Ther. 2011;13:101 10.1186/ar3204 21345260 PMC3157635

[23] 1000 Genomes Project Consortium, Abecasis GR, Altshuler D, Auton A, et al A map of human genome variation from population-scale sequencing. Nature. 2010;467: 1061–1073. 10.1038/nature09534 20981092 PMC3042601

[24] Howie B, Marchini J, Stephens M. Genotype imputation with thousands of genomes. G3 (Bethesda). 2011;1: 457–470. 22384356 10.1534/g3.111.001198 PMC3276165

[25] Mackensen F, David F, Schwenger V, et al HLA-DRB1*0102 is associated with TINU syndrome and bilateral, sudden-onset anterior uveitis but not with interstitial nephritis alone. Br J Ophthalmol 2011;95: 971–975. 10.1136/bjo.2010.187955 21059595

[26] Márquez A, Cordero-Coma M, Martín-Villa JM, et al New insights into the genetic component of non-infectious uveitis through an Immunochip strategy. J Med Genet 2017;54: 38–46. 10.1136/jmedgenet-2016-104144 27609017

[27] Rees LE, Wood NA, Gillespie KM, Lai KN, Gaston K, Mathieson PW. The interleukin-10-1082 G/A polymorphism: allele frequency in different populations and functional significance. Cell Mol Life Sci. 2002;59: 560–569. 11964134 10.1007/s00018-002-8448-0 PMC11337416

[28] Reuss E, Fimmers R, Kruger A, Becker C, Rittner C, Höhler T. Differential regulation of interleukin-10 production by genetic and environmental factors—a twin study. Genes Immun. 2002;3: 407–413. 10.1038/sj.gene.6363920 12424622

[29] Westendorp RG, Langermans JA, Huizinga TW, et al Genetic influence on cytokine production and fatal meningococcal disease. Lancet. 1997;349: 170–173. 9111542 10.1016/s0140-6736(96)06413-6

[30] Abdallah E, Waked E, Abdelwahab MA. Evaluating the association of interleukin-10 gene promoter -592 A/C polymorphism with lupus nephritis susceptibility. Kidney Res Clin Pract 2016;35: 29–34. 10.1016/j.krcp.2015.11.002 27069855 PMC4811976

[31] Barnes PF, Lu S, Abrams JS, Wang E, Yamamura M, Modlin RL () Cytokine production at the site of disease in human tuberculosis. Infect Immun. 1993;61: 3482–3489. 8335379 10.1128/iai.61.8.3482-3489.1993 PMC281026

[32] Gröndal G, Gunnarsson I, Rönnelid J, Rogberg S, Klareskog L, Lundberg I. Cytokine production, serum levels and disease activity in systemic lupus erythematosus. Clin Exp Rheumatol. 2000;18: 565–570. 11072595

[33] Llorente L, Richaud-Patin Y, Wijdenes J, et al Spontaneous production of interleukin-10 by B lymphocytes and monocytes in systemic lupus erythematosus. Eur Cytokine Netw. 1993;4: 421–427. 8186374

[34] Moore KW, de Waal Malefyt R, Coffman RL, O'Garra A. Interleukin-10 and the interleukin-10 receptor. Annu Rev Immunol. 2001;19: 683–765. 10.1146/annurev.immunol.19.1.683 11244051

[35] Van der Poll T, Marchant A, Keogh CV, Goldman M, Lowry SF. Interleukin-10 impairs host defense in murine pneumococcal pneumonia. J Infect Dis 1996;174: 994–1000. 8896500 10.1093/infdis/174.5.994

[36] Verbon A, Juffermans N, Van Deventer SJ, Speelman P, Van Deutekom H, Van der Poll T. Serum concentrations of cytokines in patients with active tuberculosis (TB) and after treatment. Clin Exp Immunol 1999;115: 110–113. 10.1046/j.1365-2249.1999.00783.x 9933428 PMC1905191

[37] Antoniv TT, Ivashkiv LB. Dysregulation of interleukin-10-dependent gene expression in rheumatoid arthritis synovial macrophages. Arthritis Rheum. 2006;54: 2711–272. 10.1002/art.22055 16947381

[38] Fathy MM, Elsaadany HF, Ali YF et al Association of IL-10 gene polymorphisms and susceptibility to Juvenile Idiopathic Arthritis in Egyptian children and adolescents: a case-control study. Ital J Pediatr: 2017 10.1186/s13052-017-0328-1 28257625 PMC5347812

[39] Zheng XY, Guan WJ, Mao C, et al Interleukin-10 promoter 1082/-819/-592 polymorphisms are associated with asthma susceptibility in Asians and atopic asthma: a meta-analysis. Lung. 2014;192: 65–73. 10.1007/s00408-013-9519-8 24162871

[40] Ewald L, Beate LW, Stephanie S, Wilfried R, Yosuf e-S. Analysis of a Functional IL-6 Gene Polymorphism in HLAB27 Associated and Intermediate Uveitis Gives New Insight in Disease Pathogenesis and Commonality with Other Autoimmune Diseases. J Immunol Res. 2015:174062 10.1155/2015/174062 26640809 PMC4657109

[41] Li H, Hou S, Yu H, et al Association of Genetic Variations in TNFSF15 With Acute Anterior Uveitis in Chinese Han. Invest Ophthalmol Vis Sci. 2015;56: 4605–4610. 10.1167/iovs.15-16896 26200500

[42] Lindner E, Weger M, Ardjomand N, Renner W, El-Shabrawi Y () Associations of Independent IL2RA Gene Variants with Intermediate Uveitis. PLoS One. 2015;10: e0130737 10.1371/journal.pone.0130737 26133380 PMC4489875

[43] Raja SC, Jabs DA, Dunn JP, et al Pars planitis: clinical features and class II HLA associations. Ophthalmology 1999;106: 594–599. 10.1016/S0161-6420(99)90122-7 10080220

[44] Tang WM, Pulido JS, Eckels DD, Han DP, Mieler WF, Pierce K. The association of HLA-DR15 and intermediate uveitis. Am J Ophthalmol 1997;123: 70–75. 9186099 10.1016/s0002-9394(14)70994-8

[45] Clive DM, Vanguri VK. The syndrome of tubulointerstitial nephritis with uveitis (TINU). Am J Kidney Dis 2018;72: 118–128. 10.1053/j.ajkd.2017.11.013 29429748

